# Weak Sinusoidal Electric Fields Entrain Spontaneous Ca Transients in the Dendritic Tufts of CA1 Pyramidal Cells in Rat Hippocampal Slice Preparations

**DOI:** 10.1371/journal.pone.0122263

**Published:** 2015-03-26

**Authors:** Kazuma Maeda, Ryuichi Maruyama, Toru Nagae, Masashi Inoue, Toru Aonishi, Hiroyoshi Miyakawa

**Affiliations:** 1 Laboratory of Cellular Neurobiology, Tokyo University of Pharmacy and Life Sciences, Hachioji, Tokyo, Japan; 2 Department of Computational Intelligence and System Science, Tokyo Institute of Technology, Midori-ku, Kanagawa, Japan; University of South Alabama, UNITED STATES

## Abstract

Neurons might interact via electric fields and this notion has been referred to as ephaptic interaction. It has been shown that various types of ion channels are distributed along the dendrites and are capable of supporting generation of dendritic spikes. We hypothesized that generation of dendritic spikes play important roles in the ephaptic interactions either by amplifying the impact of electric fields or by providing current source to generate electric fields. To test if dendritic activities can be modulated by electric fields, we developed a method to monitor local Ca-transients in the dendrites of a neuronal population in acute rat hippocampal slices by applying spinning-disk confocal microscopy and multi-cell dye loading technique. In a condition in which the dendrites of CA1 pyramidal neurons show spontaneous Ca-transients due to added 50 μM 4-aminopyridine to the bathing medium and adjusted extracellular potassium concentration, we examined the impact of sinusoidal electric fields on the Ca-transients. We have found that spontaneously occurring fast-Ca-transients in the tufts of the apical dendrites of CA1 pyramidal neurons can be blocked by applying 1 μM tetrodotoxin, and that the timing of the transients become entrained to sub-threshold 1-4 Hz electric fields with an intensity as weak as 0.84 mV/mm applied parallel to the somato-dendritic axis of the neurons. The extent of entrainment increases with intensity below 5 mV/mm, but does not increase further over the range of 5-20 mV/mm. These results suggest that population of pyramidal cells might be able to detect electric fields with biologically relevant intensity by modulating the timing of dendritic spikes.

## Introduction

Neurons in central nervous systems are exposed to endogenous extracellular electric fields [1–5]. Although synaptic transmission is considered to be the major mechanism by which neurons communicate and the electric fields merely reflect the consequences of the neural activities, there has been a notion that neurons might interact via electric fields, and such processes have been referred to as ephaptic interactions [6–9].

The intensity of extracellular electric fields in vivo was reported to be around 1–10 mV/mm [1–4, 10–12]. The threshold-intensity of fields that alter neuronal activities has been measured in various conditions, and reported to be around 1–10 mV/mm [13–16], and more recent studies reported values lower than 1 mV/mm [17, 18]. Thus, endogenous fields have enough intensity to modulate neural activities. However, it is not well understood how electric fields affect neural activities.

In this study, we attempted to examine the effects of electric fields on dendritic activities of hippocampal pyramidal neurons. Previous studies have shown that the dendrites of hippocampal pyramidal neurons are electrically excitable [19–21] because they possess various types of voltage-gated ion channels [22, 23]. Properties of dendritic spikes have been studied intensively by using brain slice preparations, and are considered to be involved in synaptic integration and neuronal plasticity. In vivo two-photon Ca imaging studies revealed ongoing transient Ca signals (Ca-transients) in the dendrites of cortical pyramidal neurons [24–27], and these signal were conjectured to be due to generation of dendritic spikes including fast Na spikes, Ca spikes and plateau potentials. However, their functional significance in physiological conditions is still unclear [21, 28].

We hypothesized that active properties of dendrites play important roles in mediating ephaptic interactions either by amplifying the impact of the electric fields or by providing current source to generate extracellular electric fields. To test if dendritic activities can be modulated by electric field, we developed a method for inducing ongoing dendritic activities and monitoring dendritic activities of a neuronal population in acute slices by modifying previously developed techniques. To define regions of interest (ROIs), we developed a novel algorithm that requires fewer assumptions than previously used algorithms [29, 30]. Using these methods, we examined the impact of weak 1–4 Hz sinusoidal extracellular electric fields on spontaneously-occurring fast Ca-transients in the distal apical dendrites of hippocampal CA1 pyramidal neurons, and found that such fields entrain dendritic activities.

## Methods

### Ethics statement

All experimental protocols followed the guidelines of the Ministry of Education, Culture, Sports, Science, and Technology of Japan and the Guide for Care and Use of Laboratory Animals (National Institute of Health, U.S.), and were approved by the Tokyo University of Pharmacy and Life Sciences Institutional Animal Care and Use Committee.

### Slice preparation

Male Wistar rats (4- to 8-week-old) (Tokyo Laboratory Animals Science, Tokyo, Japan) were anesthetized with ether and decapitated. The brain was rapidly removed and placed in cold cutting solution containing (mM): 124 choline chloride, 2.5 KCl, 1.5 MgCl_2_, 2.5 CaCl_2_, 1.25 NaH_2_PO_4_, 26 NaHCO_3_, 10 D-glucose (pH 7.4 with 5% CO_2_), then 400-μm-thick transverse hippocampal slices were cut using a vibratome (microslicer DTK-1000, Dosaka, Osaka, Japan). Before use, the slices were perfused continuously at room temperature (22–24°C) with oxygenated artificial cerebrospinal fluid (ACSF) containing (mM): 124 NaCl, 2.5 KCl, 1.5 MgCl_2_, 2.5 CaCl_2_, 1.25 NaH_2_PO_4_, 26 NaHCO_3_, 10 D-glucose (pH 7.4 with 5% CO_2_). For dye loading and experiments, the slices were transferred to a 0.5 mL submerged recording chamber and perfused with ACSF at a rate of 2–3 mL/min, and the temperature of the ACSF was held at 33 ± 1°C. During imaging sessions, ACSF was replaced with ACSF containing 50 μM 4-aminopyridine (4-AP) (ACSF/4-AP) and the KCl concentration was adjusted in the range of 3.5–7.5 mM (cACSF). All experiments were carried out in compliance with institutional guidelines for animal experiments and every effort was made to minimize the number of animals used.

### Dye loading

**Fig 1:**
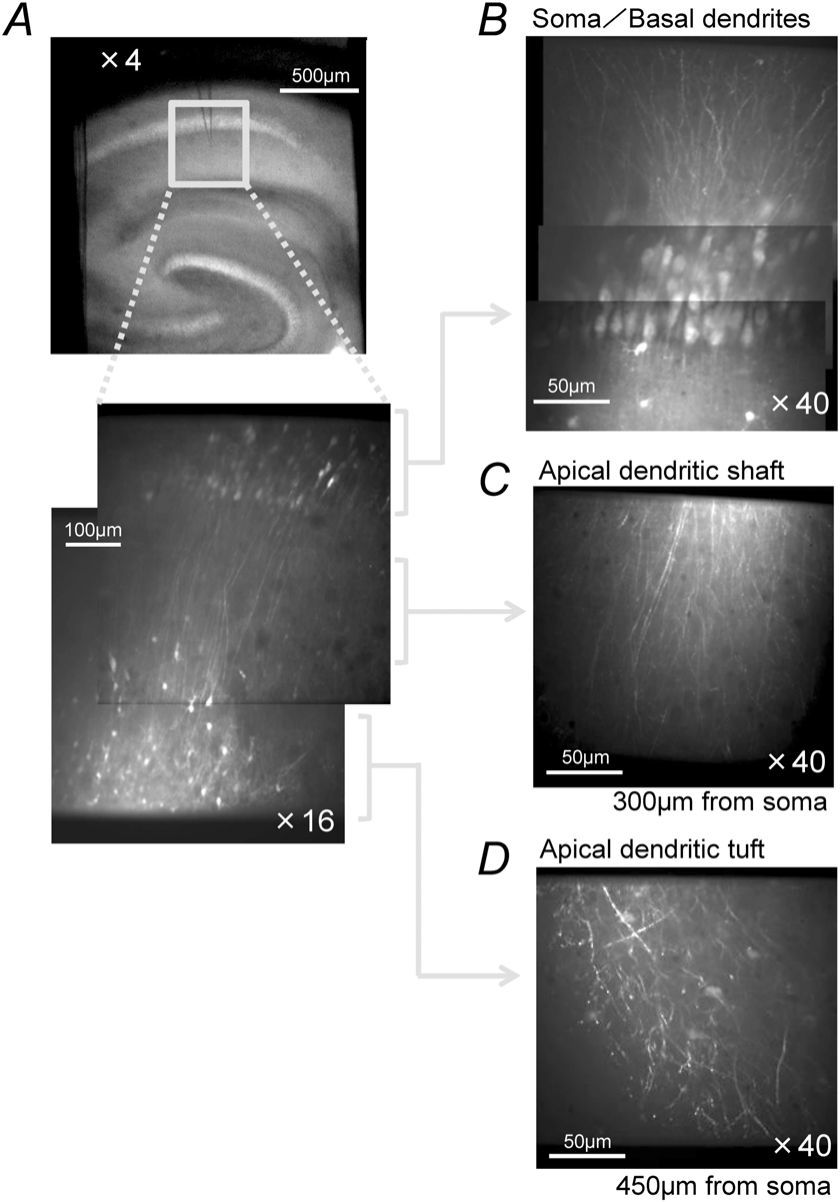
CA1 pyramidal neurons loaded with Oregon Green 488 BAPTA-1. Acute hippocampal slices (400 μm thick) were prepared from 4- to 8-week-old Wister rats and a slice was placed in a submerged type recording chamber mounted on the stage of an upright microscope equipped with a confocal microscopy system. (*A*), Top panel: The tip of a glass pipette filled with buffer solution containing 500 μM Oregon Green 488 BAPTA-1 AM was positioned in the str. radiatum 50 μm below the surface and 90 μm from the cell layer, and then a pressure of 70 kPa was applied for 40–50 minutes to eject the dye-containing solution from the pipette. After loading, the pipette was removed. Bottom panel: A confocal fluorescence image at low magnification (x16 objective lens). Note that many dendrites and non-pyramidal neurons are visible in the distal part of the str. radiatum and str. lacnosum-moleculare. (*B*)*-*(*D*) Confocal fluorescence images at a higher magnification (x40 objective lens). The soma and basal dendrites (*B*), tuft of the apical dendrites (*C*) and the shaft of the apical dendrites (*D*) of multiple neurons are loaded with the dye.

The multi-cell dye loading technique [31–33] was used to label the apical dendrites of neurons in the hippocampal CA1 area with a fluorescent Ca-indicator, Oregon Green 488 BAPTA-1 AM (OGB-1AM; Invitrogen, Carlsbad USA). A 5 mM stock solution of OGB-1AM was prepared by dissolving 50 μg of OGB-1AM in 20% Pluronic F-127 (Invitrogen) in dimethylsulfoxide, which, before use, was diluted tenfold in external buffer (150 NaCl mM, 2.5 KCl mM, 10 HEPES, mM), sonicated for 1 minute, and filtered. A glass pipette filled with 1.5 μL of dye solution was connected by tubing to a pressure gauge and a 10 ml syringe through a three way stopcock. The tip of the pipette was positioned under visual guidance using infra-red differential contrast (IR-DIC) microscopy in the striatum (str) radiatum 10–20 μm from the somatic layer of the CA1 region and inserted obliquely into the slice preparation at an angle of 30° (Fig.1A) to the surface in a direction parallel to the apical dendrites till the tip of the pipette was 50 μm below the surface. Then a pressure of 70 kPa was applied for 40–50 minutes to eject dye solution from the pipette. All dye loading steps were performed in the recording chamber at 32–37°C under continuous perfusion with ACSF. After loading, the pipette was removed.

### Ca imaging

To monitor the change in fluorescence intensity of the Ca-indicator, a fast confocal microscopy system (CSU-10; Yokogawa, Kanazawa, Japan) attached to an upright microscope (E600FN; Nikon, Tokyo, Japan) was used. A hippocampal slice preparation was placed in a submerged recording chamber mounted on the stage of the microscope and viewed with a water immersion objective lens (X40, N.A.0.8, Nikon or X16, N.A. 0.80, Nikon). The CSU-10 is based on a Nipkow spinning-disk scanner, which has two disks, one with a microlens array and the other with pinholes arranged in the same array which both rotate at 1,800 rotations per minutes. The 488 nm peak of an Ar-Kr gas laser (MELLES GRIOT, Albuquerque, USA) giving 50 mW power output was used as the light source. The fluorescence from the specimen was reflected by a dichroic mirror (reflects > 500 nm) located between the rotating disks, optically filtered with a high cutoff filter (500 nm), and projected onto the image plane of an electron multiplying CCD camera (ImagEM, C9100-13, HAMAMATSU, Hamamatsu, Japan). The camera has an image plane of 512 x 512 pixels and digitizes the signals from each pixel at 16-bit resolution, and is capable of taking full-frame images at a rate of 31.9 Hz; however, in most of the recordings in this study, the frame rate was increased to 60.9 Hz (16.4 msec frame interval) by performing 2 x 2 binning. Image data were acquired using AQUACOSMOS software (HAMAMATSU) running on a PC and stored as binary files, which were analyzed off line using in-house generated software.

### Application of sinusoidal electric field

**Fig 2:**
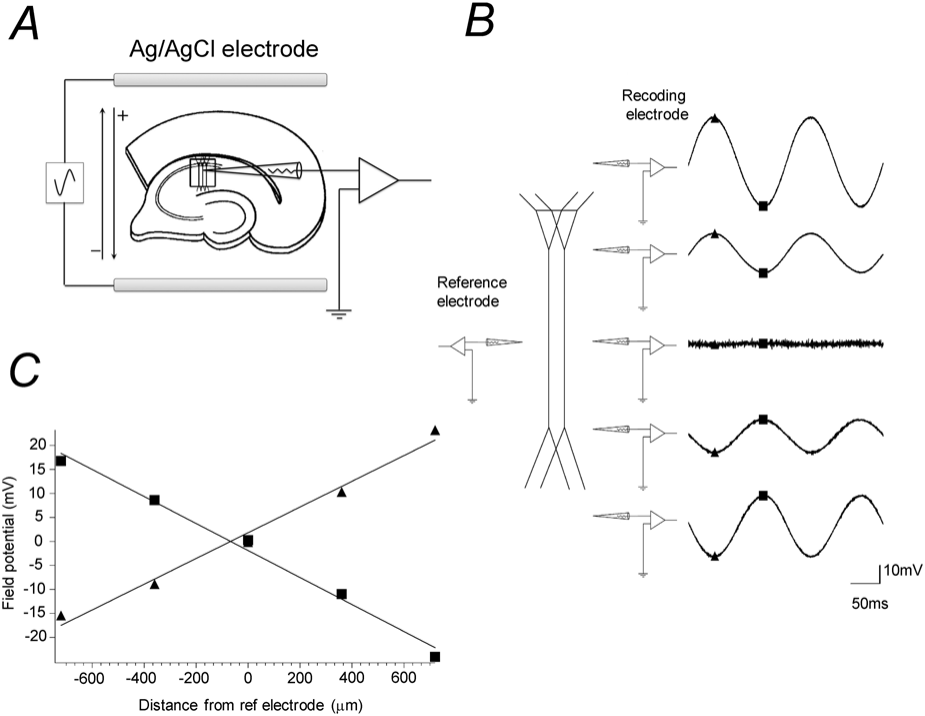
Sinusoidal electrical field stimulation. (*A*) Electric current was applied with a pair of Ag/AgCl electrodes placed 5 mm apart in parallel on the bottom of a submerge type recording chamber. A piece of hippocampal slice preparation was placed between the electrodes. A sinusoidal wave form was generated using in-house developed software run on a PC with a 12-bit digital-to-analog converter. The voltage output was electrically isolated and converted to current using a voltage-to-current converter. (*B*) Examples of extracellular field potential recorded from the CA1 area of a slice preparation. Field potential was recorded at 360 μm interval across the CA1 region from the basal to apical dendrite during the passage of 6 Hz sinusoidal current with a glass pipette inserted into the tissue of the slice. The potential was measured with respect to a reference electrode placed at the middle of str. radiatum throughout the recording. (*C*) Peak values of extracellular field potential at the top and bottom of the stimulation plotted against the location of the electrode. With our setup arrangement, the field intensity was uniform along the somato-dendritic axis and was independent of whether the recording electrode was inserted into the slice or was placed outside of the slice. The peak intensity of the sinusoidal electric field stimulation was 27.8 mV/mm in this particular case. Unless specified otherwise, the frequency of the sinusoidal wave was 4 Hz (more precisely 3.79 Hz or 3.95 Hz).

To generate the electric field, two parallel 15 mm long Ag/AgCl electrodes were placed 5 mm apart on the bottom of the recording chamber and a hippocampal slice was placed between the electrodes with the lamina of the CA1 field aligned parallel to the electrodes (Fig. 2). With this alignment, the direction of the electric field was parallel to the somato–dendritic axis of the CA1 pyramidal neurons. To apply sinusoidal electric field, a sinusoidal wave form was generated using in-house developed software run on a PC equipped with a 12-bit digital-to-analog converter. The voltage output was electrically isolated and converted to current using an in-house made voltage-to-current converter in which the current was supplied from a pair of 12 volt car batteries. The applied current generates electric field in a direction perpendicular to the electrodes.

Fig. 2B shows an example of extracellular field potential recorded from the CA1 area of a slice preparation during 6 Hz sinusoidal current application. Field potential was recorded at 360 μm interval across the CA1 region perpendicular to the str. pyramidale, along the somato-dendritic axis of the pyramidal cells, with respect to a reference electrode placed at the middle of str. radiatum. The intensity of the resultant electric field was calculated as the gradient of the field potential (Fig. 2C). With our electrode alignment, the field intensity was uniform along the somato-dendritic axis. In the measurement shown in Fig. 2, the tip of the electrode was inserted into the tissue of slice preparation. The potential was the same whether the tip of the recording electrode was placed in the slice tissue or was placed out of the tissue because most of the stimulating current flow outside of the slice tissue. To determine appropriate current intensity to apply electric fields with intended intensity, intensity of electric field was measured by recording bath potential with a glass pipette at three locations within the space between the Ag/AgCl electrode pair before placing slice preparations. The frequency of the sinusoidal field was set to be 4 Hz (more precisely 3.7 Hz or 3.95 Hz) unless specified otherwise.

### Algorithm for defining cell structures from Ca imaging data

To isolate signals from different structures, we developed a novel algorithm using non-negative matrix factorization (NMF) [34]. Using numerical simulations, we confirmed that, in certain parameter ranges, this method detects regions of interests (ROIs) with a higher accuracy than the PCA-ICA algorithm [29]. A more detailed explanation and the evaluation of this method are presented elsewhere [35]. Here, we briefly summarize the algorithm.

We assume that a movie data matrix ***F*** (*N* pixels by *T* time frames) is the summation of the specific fluorescence signals from *K* cells, background fluorescence, and noise. Since ROIs are stationary, the problem can be thought of as decomposition of the data matrix ***F*** into two low rank matrices, the spatial component ***A*** (*N* x *K*, the *k*th-column vector representing the shape of the *k*th cell) and the temporal component ***S*** (*K* x *T*, the *k*th row vector representing the time series of the *k*th cell). In addition, the data matrix contains a background component expressed as the product of the spatial intensity distribution ***a***_***b***_ and the time course ***s***_***b***_. Thus we have the generative model:

$$F=AS+a_{b}s_{b}+noise.$$

ROIs can be detected by estimating the matrices ***A***, ***S***, and ***a***_***b***_.

In the imaging data, horizontal and vertical stripes appear as artifacts as a consequence of using spinning-disk confocal microscopy. These artifacts interfere with the ROI determining algorithm, so were removed by low-pass filtering the image sequence in the pre-processing step. Stripe shaped artifacts due to the rotation of the spinning disks appeared as sharp peaks in the temporal power spectrum, so the cutoff frequency of the low-pass filter was set to remove these artifact-derived peaks. The bleaching rate of fluorescence was then estimated from the pre-processed data and the bleaching line, ***s***_***b***_, was given by a decreasing linear function with the bleaching rate.

Assigning the pre-processed data to the data matrix ***F*** and using the bleaching line ***s***_***b***_ given a priori, the objective function was optimized using the modified version of the alternating least squares algorithm [34] and the decomposed matrices and vectors ***A***, ***S***, and ***a***_***b***_ estimated. After optimization, we manually selected the column vectors of ***A*** that apparently capture part of cells and obtained ROIs capturing the contours of part of the cells by binarization of the selected vectors. The reasons for manual selection and binarization are that this algorithm sometimes detects erroneous components.

We detected the rising phases of Ca-transients using following steps: 1) smooth the time series at the selected ROIs by taking a moving average, 2) calculate the temporal differentiations of the smoothed time series, i.e., ***ds*(t) = *s*(t)—*s*(t-1)**, and 3) identify time points where ***ds*(t)** rises above a certain threshold. The threshold was selected as twice the standard deviation of the noise.

### Spiral plot

To visualize the phase of the Ca-transients during application of a sinusoidal field, we generated diagrams which we refer to as spiral plots (Fig. 6G, 7). The onset of all the Ca-transients recorded from all the ROIs was plotted on a spiral line in the x-y plane. The spiral line is a time axis that starts from a point on the x-axis and rotates counter clockwise at the angular velocity of the sinusoidal stimulating field. The radius of the rotation gradually increases as time proceeds. Different colors were assigned to each ROI and the onsets of the Ca-transients recorded from the same ROI were marked on the time axis as bars of the same color as the ROIs. To compare the phases of the Ca-transients generated during the period with no field stimulation with those generated during stimulation, a similar diagram was generated for the period with no stimulation using the angular velocity of the sinusoidal field used for stimulation. The phase of the spiral plot corresponds to the phase of the sinusoidal field. In our convention, the 0–180° phase is the phase in which the extracellular current flows from the basal dendrites towards the apical dendrites. During this phase, the membrane potential of the soma is hyperpolarized, and the distal apical dendrites depolarized (Fig. 6D).

### Circular statistics

In order to quantify the distribution of the phase and define the mean phase to which fast-Ca-transients are locked, we used techniques for handling circular data [36]. The axis of the spiral plot was shrunk to a simple circle with unit radius, and each of the bars marked on the circle was considered to represent a unit vector. Summation of the vectors gives rise to a resultant vector $\vec{R}$, the direction of which represents the *mean phase* (or *mean direction*) $\bar{\theta }$ of the unit vectors. By dividing the length of the resultant vector by the number of component unit vectors, we obtain a quantity referred to as the *mean resultant length $\bar{R}$*_._ To quantify the extent of entrainment (phase-locking) in a manner appropriate for circular data, a quantity referred to as the *circular standard deviation cSD* for the von Mises distribution [37] was used. *cSD* is defined as follows.

$$cSD\;=\;{\left[-2\log\left(\overset{-}{R}\right)\right]}^{1/2}$$

To quantify the extent of entrainment in terms of angular degree, we defined the c*ircular standard distribution in terms of the angular degree* (*acSD*) by multiplying the *cSD* by 180°/π.

The Rayleigh test [36] was used to test the uniformity of the circular distribution of fast-Ca-transients. A null hypothesis of uniform circular distribution will be rejected if $\bar{R}$ is too large. We calculate significance probability of the null hypothesis as follows [38].

$$P\;=\;exp\left[\sqrt{\left(1+4N+4N^{2}\left(1-{\left(\overset{-}{R}\right)}^{2}\right)\right)}-\left(1+2N\right)\right]$$

Here *N* represents the number of transients.

## Results

### Spontaneous Ca-transients recorded from the dendrites

We simultaneously monitored Ca-transients from the dendritic branches of many neurons. Fig. 1 shows the fluorescence images of the dye-filled cells in the CA1 field in acute hippocampal slices (Fig. 1A). The basal dendrite (Fig. 1B), the shaft of the apical dendrites (Fig. 1C), and the tuft of the apical dendrites (Fig. 1D) of pyramidal neurons can clearly be identified. Based on the location of the soma and the shape of the neurites, the majority of dye-labeled cells were identified as pyramidal neurons, while small number of cells were identified as non-pyramidal neurons, likely be either interneurons or glial cells.

**Fig 3:**
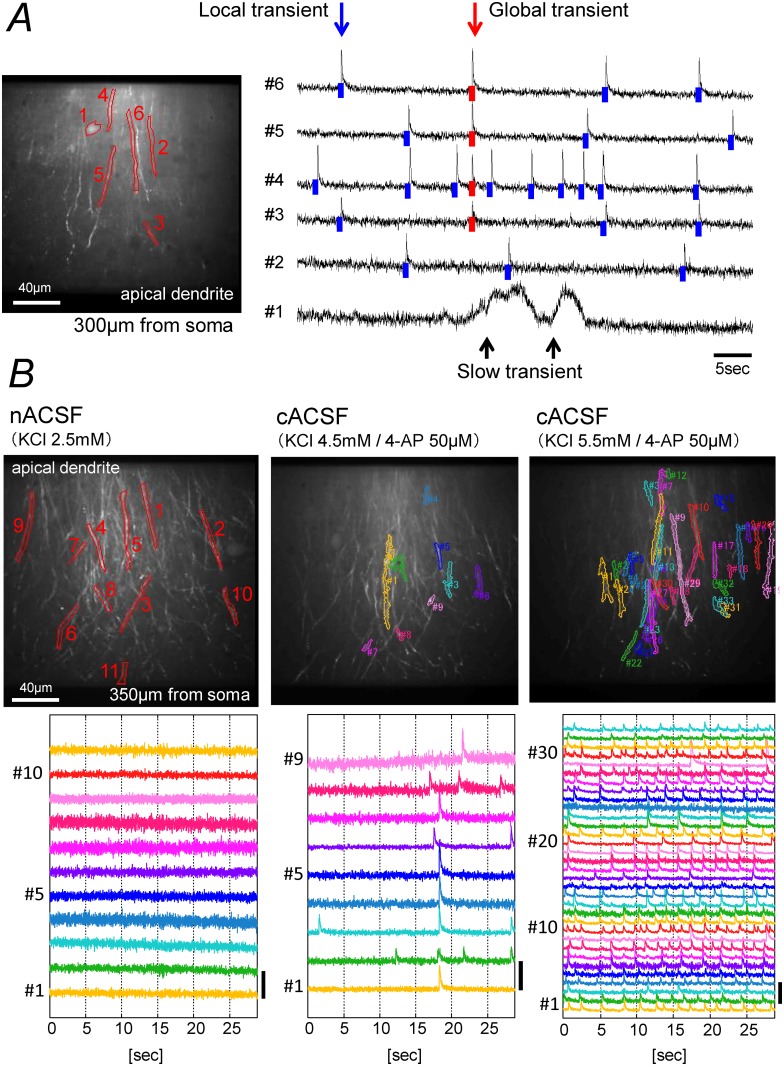
Typical Ca fluorescence signals recorded from apical dendrites. (*A*) Three types of spontaneous Ca-transient seen in ACSF/4-AP containing 3.75 mM K^+^ ion. Left panel: The field of view. The center of the image field was 300 μm from the border between the cell layer and the str. pyramidale. Six ROIs were selected by drawing lines around brightly stained neurites. Right panel: Time-course of the fluorescence intensity of the ROIs shown in the left panel. A round shaped cell (ROI #1) showed slow-transients, while dendritic branches (ROI #2–6) showed fast-transients. The transients marked with blue bars are local-fast-Ca-transients, while those marked with red bars are global-fast-Ca-transients. (*B*) Fast-Ca-transients in ACSF with different composition. The recordings were made from the same field with a slightly altered focus. In the middle and right panels, the ROIs were determined using the NMF-based algorithm and the data for all ROIs reported by the NMF-based algorithm are shown. Left panels: Responses in standard ACSF containing 2.5 mM K^+^ ion. No Ca-transient was detected during this session. Hence the ROIs were selected by drawing lines around brightly stained neurites by eye. Scale bar (ΔF/F): 10%. Center panels: In ACSF/4-AP containing 4.5 mM K^+^ ion. During the 30 sec recording session, 9 ROIs showed fast-Ca-transients. Scale bar (ΔF/F): 10%. Right panels: In ACSF/4-AP containing 5.5 mM K^+^ ion. Fast-Ca-transients were detected from 32 ROIs. Scale bar (ΔF/F): 20%.

Fig. 3A shows typical spontaneous Ca-transients in the str. radiatum of the CA1 field recorded in ACSF/4-AP containing 3.75 mM K^+^ ion. In this condition, we noted that the Ca-transients could be classified into two types, fast and slow. The fast-Ca-transients were further classified as local or global. The fast-Ca-transients rose quickly, peaking within a few frames, then rapidly decayed back to the baseline level. The slow-Ca-transients rose slowly to a maximum level in a few seconds, and then slowly decayed back to the baseline level with a half-maximal width of 2.2–6.6 sec (10 slices, 10 ROIs, 10 transients). Some of the fast-Ca-transients showed strong synchronization, as almost all of the pixels were synchronized within the limits of our frame interval of 16.4 msec. We classified these as global-fast-Ca-transients and the rest of the fast-transients as local-fast-Ca-transients.

In normal ACSF, the soma and dendrites of the dye-stained neuron rarely showed Ca-transients (Fig. 3B left panel). Because our objective was to examine the impact of weak electric fields on Ca-transients in the dendrites, it was necessary to find conditions in which the dendrites show ongoing activity similar to that seen in physiological conditions in vivo. To mimic situations in vivo in which the extracellular K^+^ concentration is higher due to ongoing activities and neurons receive incessant endogenous neuro-modulatory inputs, we adjusted extracellular K^+^ concentration and added low concentration of 4-AP to the bathing solution. We found that the dendrites of pyramidal neurons show spontaneous Ca-transients when the K^+^ concentration was increased from 2.5 mM to 3.5–7.5 mM and 50 μM 4-AP was added to the ACSF (Fig. 3B, center and right panels). We adjusted K^+^ concentration within the range of 3.5–7.5 mM so that the dye-loaded neurons constantly show many local-fast-Ca-transients and few global-fast-Ca-transients. Higher concentrations of K^+^ ion (> 7.5 mM) were avoided, as they caused swelling of the slice preparations.

The Ca-transients shown in Fig. 3A were signals obtained from manually determined ROIs. With manually determined ROIs, some of the ROIs gave a mixture of signals from different cellular elements, such as the soma of one neuron and the dendrites of another neuron. To isolate Ca-transients from separate cells or separate parts of the same cell, we determined ROIs by applying a novel algorithm we devised based on NMF. Using this algorithm, separate parts of the same neuron may constitute separate ROIs that generate separate signals. In this study, we analyzed isolated signals obtained using this algorithm. Of the total number of Ca-transients, 94% were fast-Ca-transients, most (86%) of which were local-fast-Ca-transients, while only 6% of the total number of transients were slow-Ca-transients. The shapes of the ROIs that exhibited slow-Ca-transients were that of the soma of round cells or that of cells with a large soma and a rich dendritic arborization located in the str. radiatum. It is likely that these cells are either interneurons or glial cells. The half-maximal width of 2.2–6.6 sec of slow-Ca-transients is consistent with the characteristics of Ca signals reported for glial cells [39–41]. In this study, we focused on the fast-Ca-transients recorded from the dendrites of pyramidal neurons.

### Characteristics of fast-Ca-transients

**Fig 4:**
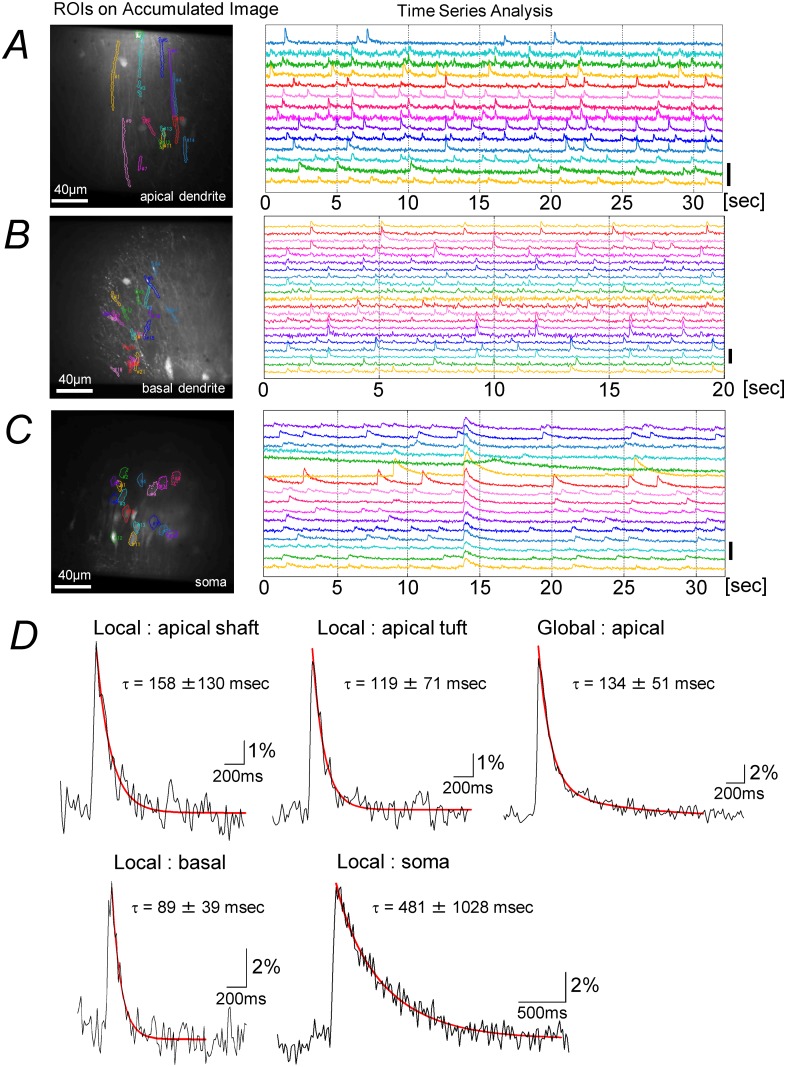
Spontaneous fast-Ca-transients recorded from different parts of a pyramidal neuron. (*A*) Distal part of the apical dendrites. The center of the image field was 300 μm from the border between the cell layer and the str. pyramidale. The cASCF contained 7.5 mM K^+^ ion. (*B*) Basal dendrites. The cACSF contained 6.5 mM K^+^ ion. (*C*) Soma. The cACSF contained 3.0 mM K^+^ ion. All the ROIs in panel (*A*)-(*C*) were determined using the NMF-based algorithm. Frame rate 16.4 msec. Time course of the fluorescent intensity recorded at the ROIs shown on the left panels with different colors are shown on the right panels using the same colors. Scale bar (ΔF/F): 20%. (*D*) Comparison of the decay time constant (τ) of the fast-transients from different locations. Frame interval: 16.4 msec. Data for all fast-Ca-transients from a representative recording trial recorded at respective locations of each slice preparation were digitally averaged. For respective locations, 4 ROIs were randomly selected from each of the trials, and the decay time constants were obtained from the averaged traces. The decay phase of the traces was fitted to a double exponential function, and the values for the shortest time constants are shown.

Fig. 4 shows spontaneous fast-transients recorded from the apical dendrite (*A*), basal dendrite (*B*), and the soma (*C*) of pyramidal neurons. As shown in Fig. 4D, the fast-Ca-transients rose quickly, peaking within a few imaging frames (10–90% rise time of local-fast-Ca-transients: apical tuft 29.6 ± 5.5 msec (3 slices, 10 ROIs, 150 transients), apical shaft 27.5 ± 5.1 msec (4 slices, 14 ROIs, 228 transients), soma 35.2 ± 6.7 msec (4 slices, 14 ROIs, 224 transients), basal dendrite 27.9 ± 6.1 msec (4 slices, 9 ROIs, 143 transients); global-fast-Ca-transients: 26.0 ± 2.2 msec (4 slices, 7 ROIs, 76 transients)). The decay time constants depended on the location. While fine dendrites showed fast time constants (apical tuft 119 ± 71 msec (3 slices, 10 ROIs, 41 transients), basal 89 ± 39 msec (3 slices, 12 ROIs, 47 transients)), thicker dendrites and the soma showed slower time constants (apical shaft 158 ± 130 msec (4 slices, 14 ROIs, 50 transients), soma 481 ± 1028 msec (4 slices, 16 ROIs, 70 transients)).

**Fig 5:**
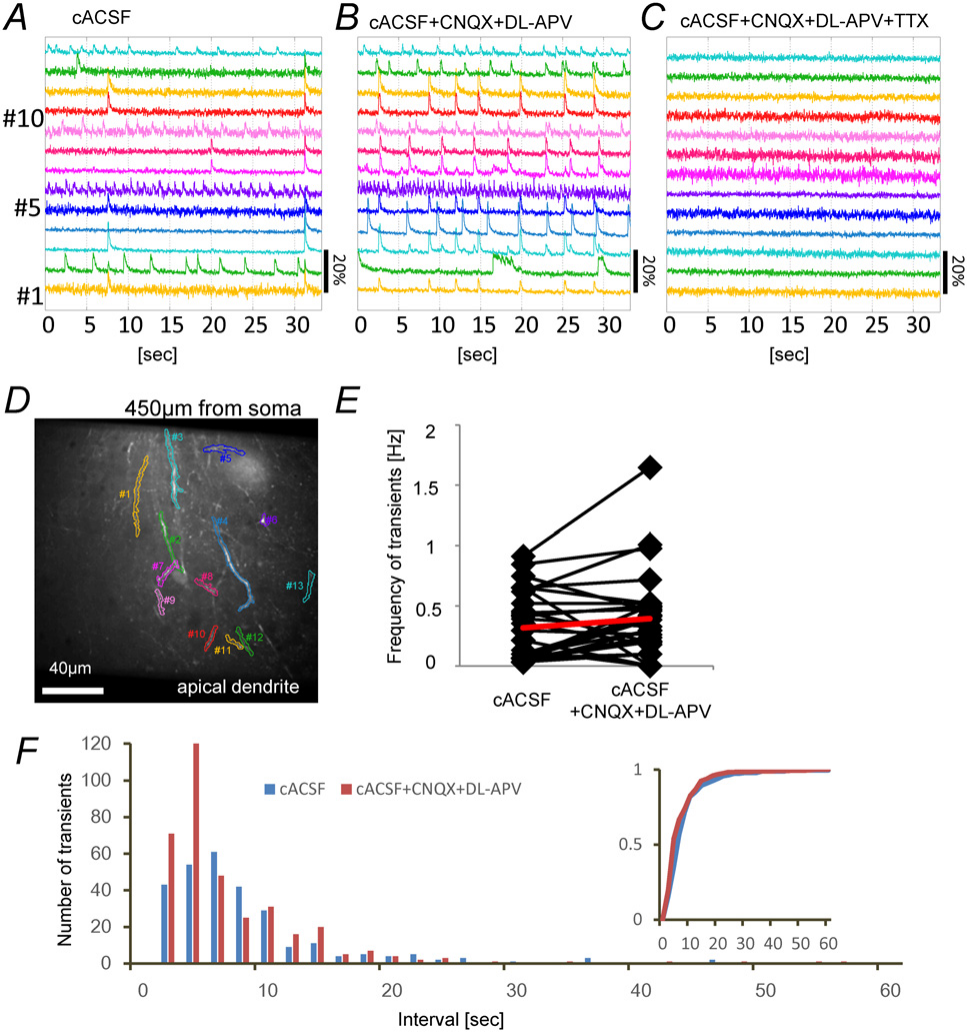
Pharmacological characterization of spontaneous fast-Ca-transients. (*A*)—(*C*) Fast-Ca-transients recorded from the distal apical dendrites of the same preparation. (*A*) In cACSF with 3.5 mM K^+^ ion. (*B*), In cACSF containing 10 μM CNQX and 50 μM DL-APV (*C*). In the presence of 1 μM TTX added to the solution used in (*B*). Scale bar (ΔF/F): 20%. The recordings in (*B*) and (*C*) were made 15 minutes after switching the solution. (*D*) ROIs for panels (*A*)**—**(*C*). To allow comparison, the same ROIs were used throughout. The center of the image field was 450 μm from the border between the cell layer and the str. pyramidale. (*E*) Frequency of the fast-Ca-transients in 24 ROIs in the absence or presence of CNQX and DL-APV; the red line shows the mean values. There was slight increase in frequency in the presence of CNQX and DL-APV (153 ± 256%), but the change was not significant (Wilcoxon signed-rank test P>0.05, 4 slices, 24 ROIs). (*F*) Interval histogram of fast-Ca-transients in 24 ROIs in the absence or presence of CNQX and DL-APV constructed using the same set of data used for panel *E*; the blue bars show number of transient-intervals in cACSF, red bars show number of transient-intervals in the presence of CNQX and DL-APV (bin width: 2 sec). Inset shows the cumulative fraction of transient-intervals.

Fig. 5A shows Ca-transients recorded from an area more than 350 μm distant from the str. pyramidale, near the border between the str. radiatum and str. lacnosum-moleculare. All the ROIs delineated and numbered in Fig. 5D showed fast-Ca-transients in cACSF. The frequency of the transients was high in some of the elements (ROIs. #2, #6, #9 and #13) and low in the others (ROIs. #1, #3–5, #7, #8, and #10–12). Because the cACSF contained 50 μM 4-AP and a higher K^+^ concentration than the standard ACSF, it was conceivable that the pyramidal neurons in the CA3 field generated bursts of action potentials and provided excitatory synaptic inputs to trigger Ca-transients in the dendrites of CA1 neurons. However, in the presence of a mixture of CNQX (10 μM) and DL-APV (50 μM), blockers of AMPA-type and NMDA-type glutamate receptors respectively, the fast-Ca-transients were not abolished (Fig. 5B). The distribution of transient-intervals (Fig. 5F) and the frequency of the transients (Fig. 5E) was not significantly altered (Wilcoxon signed-rank test, p> 0.05; 4 slices, 24 ROIs) by applying CNQX and DL-APV, showing that the fast-Ca-transients were not due to excitatory synaptic inputs.

The fast-Ca-transients were completely abolished (4 out of 4 slices, 24 ROIs) after further addition of TTX (1 μM) to the cACSF containing 10 μM CNQX and 50 μM DL-APV (Fig. 5C), showing that they are likely to be due to the generation of Na-spikes in the dendrites and not to the generation of Ca-spikes or Ca-release from internal stores.

### Effects of extracellular sinusoidal electric fields on dendritic local-fast- Ca-transients

**Fig 6:**
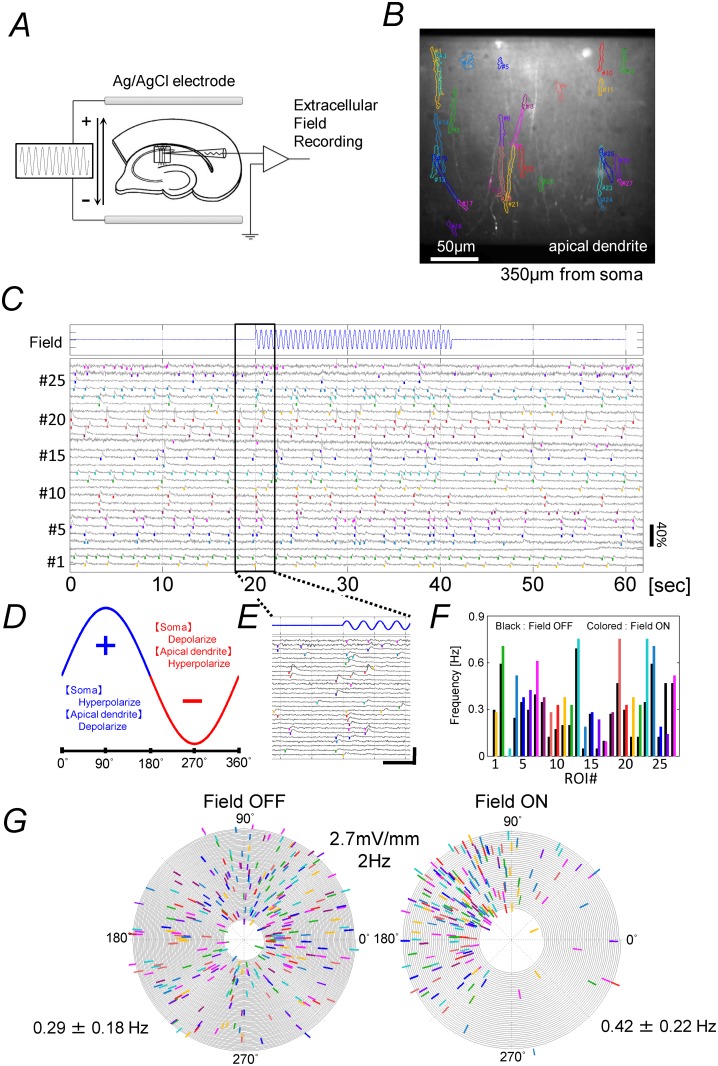
Analysis of the impact of sinusoidal extracellular electric fields on fast-Ca-transients. (*A*) Arrangement of the electrodes for field stimulation. Electric current was applied between a pair of Ag/AgCl electrodes to generate electric field. The intensity of the field was monitored by measuring bath potential. (*B*) ROIs for the data shown in (*C*). The center of the image field was 350 μm from the border between the str. pyramidale and str. radiatum. (*C*) Representative data showing fast-Ca-transients during sinusoidal field stimulation. After recording Ca signals for 20 sec in ACSF/4-AP containing 7.5 mM K^+^ ion, field stimulation (2.7 mV/mm, 2 Hz (1.88 Hz), 20 cycles)was turned on for 20 sec, then off for further 20 sec while recording the signals. Scale bar (ΔF/F): 40%. The onsets of fast-Ca-transients are marked with bars of the same color as the ROIs shown in (*B*). (*D*) Direction of the stimulating current and the phase. (*E*) Expanded traces of the signals near the onset of stimulation. (*F*) Frequency of Ca-transients during the periods in which the stimulation was turned on (colored bars, mean frequency 0.42 ± 0.22 Hz) or turned off (black, mean frequency: 0.29 ± 0.18 Hz) for ROIs indicated by the same colors in (*B*). (*G*) Spiral plot of the data shown in (*C*). The right panel shows data during the period in which the field was applied, and the left panel for the period with no field. The same angular velocity was used for both panels. Note that the colored bars are mainly located within the range of 60–240° during field stimulation.

To examine the impact of weak electric fields on local-fast-Ca-transients, we applied sinusoidal electric fields parallel to the soma-dendritic axis of CA1 pyramidal neurons (Fig. 6A) and analyzed the frequency, timing, and synchronization of fast-Ca-transients in the distal apical dendrites. In previous studies [42, 43], we have shown that a rectangular extracellular current pulse applied parallel to the somato-dendritic axis in the direction from the basal to apical dendrites gives rise to a change in membrane potential that reverses the polarity at locations 160–250 μm from the str. pyramidale: the soma and basal dendrite hyperpolarize, while the distal apical dendrites depolarize. We therefore examined the impact of an electric field in the distal part of the str. radiatum and str. lacnosum-moleculare more than 350 μm distant from the str. pyramidale, because the polarity of the change in membrane potential during electric field stimulation should be the same within this area.

Using a pair of bar-shaped Ag/AgCl electrodes, we applied sinusoidal electric fields by supplying current across a slice preparation (Fig. 2, 6A, 6D). The intensity and phase of the fields were monitored by recording the extracellular potential with a glass electrode placed in the str. radiatum. The frequency range of the applied field was 1–4Hz, and the range of the field intensity was 0.5–25.0 mV/mm (peak values). In a representative experiment shown in Fig. 6, after recording spontaneous Ca signals for a period of 20 seconds, a sinusoidal current with a frequency of 2 Hz, intensity of 2.7 mV/mm was applied for 20 seconds, and then spontaneous signals were again recorded for 20 seconds. Fig. 6C shows the time course of the dye signal (ΔF/F) from all 27 ROIs isolated using our NMF-based algorithm within the field of view, which was 350 μm from the str. pyramidale. The colored bars marked on the traces (Fig. 6C, 6E) indicate the onset of fast-Ca-transients recorded from the ROIs shown in the same color (Fig. 6B). The frequency of the fast-Ca-transients was slightly higher during field stimulation (Fig. 6F, 6G).

We compared the timing of local-fast-Ca-transients during field stimulation with those during the period of no stimulation. For this purpose, we visualized the timing of Ca-transients by making spiral plots (Fig. 6G) during the period in which no field was applied (left panel; Field OFF) and during the field stimulation (right panel; Field ON). The spiral plot shows that the timing of the onset of fast-Ca-transients during application of the sinusoidal field in terms of angular degree was unevenly distributed, being mostly within the 60–240° range, whereas, with no field, the timing was evenly distributed over the entire phase. Thus, the activities of the distal apical dendrites of the pyramidal neurons in this preparation became entrained to the extracellular sinusoidal electric fields at a field intensity of 2.7 mV/mm.

### Relations between field intensity and local-fast-Ca-transients

**Fig 7:**
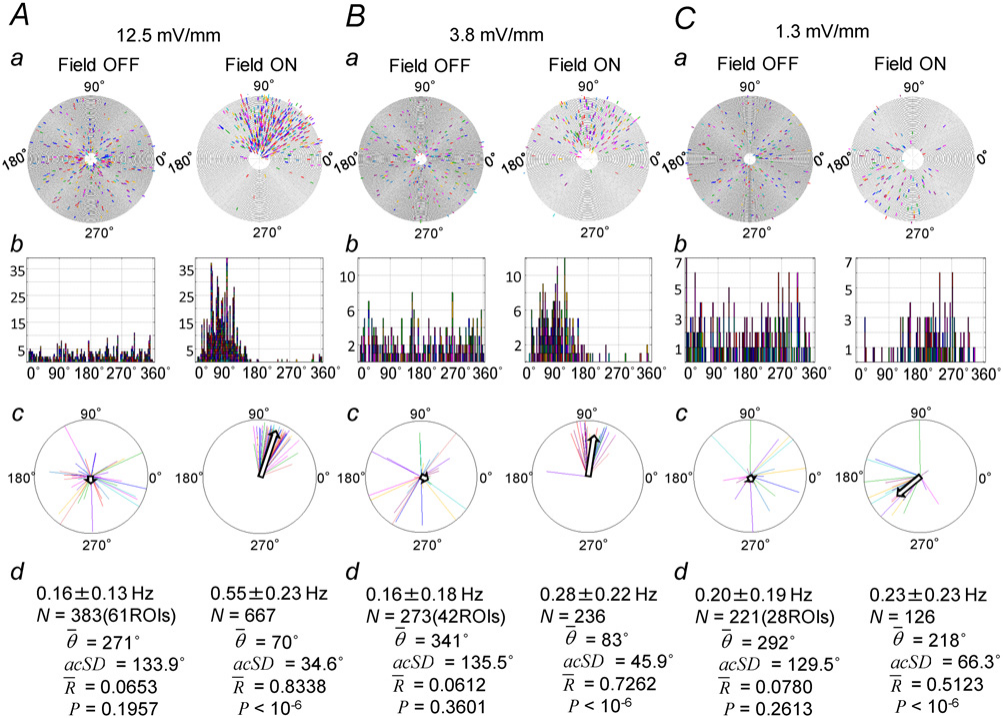
Entrainment of fast-Ca-transients at different field intensities. (*A*,*B*,*C*) 4 Hz sinusoidal electric field of three different intensities was applied to the same slice preparation in ACSF/4-AP containing 6.5 mM K^+^ ion. Image recording was made from the distal apical dendrites with a frame interval of 16.4 msec. Analysis of data obtained in experiments with a field intensity of 12.5 mV/mm (*A*), 3.8 mV/mm (*B*), or 1.3 mV/mm (*C*) are shown. Top panels (*a*): Spiral plots. The right panels show data recorded during the period in which the field stimulation was applied (Field ON) and the left panels for the period with no applied field (Field OFF). Note that the fast-Ca-transients were entrained to the applied field even at the field intensity of 1.3 mV/mm. Upper middle panels (*b*): Phase distribution histograms for the data shown in (*a*). Bin width 3°. The vertical axis represents number of fast-Ca-transients for the bins of the histogram. The horizontal axis represents the phase in terms of angular degree. Lower middle panels (*c*): Circular diagrams constructed for the same set of data showing mean resultant vectors. The colored lines represent the mean resultant vectors for the Ca-transients recorded from each of the ROIs and the arrows represent the mean resultant vector for the Ca-transients recorded from all the ROIs (see Methods). Bottom panels (*d*): Statistical values for all the fast-Ca-transients recorded from all the ROIs; frequency of the Ca-transients, total number of the transients (*N*), mean phase ($\bar{\theta }$), circular standard deviation in terms of angular degree (*acSD*), mean resultant length ($\bar{R}$), and the significance probability for the null hypothesis of uniform circular distribution (*P*).

Fig. 7 shows three pairs of spiral plots and the statistical analysis for data obtained from the same preparation using 4 Hz sinusoidal electric fields of different intensities; 12.5 mV/mm (*A*), 3.8 mV/mm (*B*), and 1.3 mV/mm (*C*). In all three cases, the fast-Ca-transients were entrained to the field. The entrainment was more easily seen at the field intensity of 12.5 mV/mm than at weaker intensity. Interestingly, the phase of the transients seemed to distribute within the same width of phase in all three cases. To analyze the extent of entrainment, we plotted phase distribution histograms (Fig. 7A-C, upper middle panels (*b*)), which showed that the phase distribution was uniform when there was no field, but was clustered during field stimulation. The lower middle panels (*c*) in Fig. 7A-C show the circular diagrams constructed for the same set of data, showing the mean phase $\bar{\theta }$, the mean resultant vector (colored lines) calculated from the transients recorded for each ROI, the mean resultant vector *$\vec{R}$* (arrows) calculated from all the transients obtained from all the ROIs. Statistical values are listed in bottom panels (*d*). For all three values of field stimulation intensity, the mean resultant length $\bar{R}$ during field stimulation was greater than that during the period with no field. The significance probability *P* for the Rayleigh test with a null hypothesis, that the circular distribution of Ca-transients is uniform, were large during the period with no electric fields, while the values were smaller than 10^-6^ during the period with field, implying that that the distribution was not uniform when the sinusoidal field was present and that the electric fields indeed entrained the local-fast-Ca-transients.

The decay time constant of fast-Ca-transients during field stimulation recorded from the apical tuft of the dendrites was 124 ± 50 msec (3 slices, 10 ROIs, 20 transients), and was not significantly different from that during the period with no field (P = 0.364, Student's t-test), suggesting that the change in the phase of the Ca-transients was not accompanied by change in the underlying activity.

**Fig 8:**
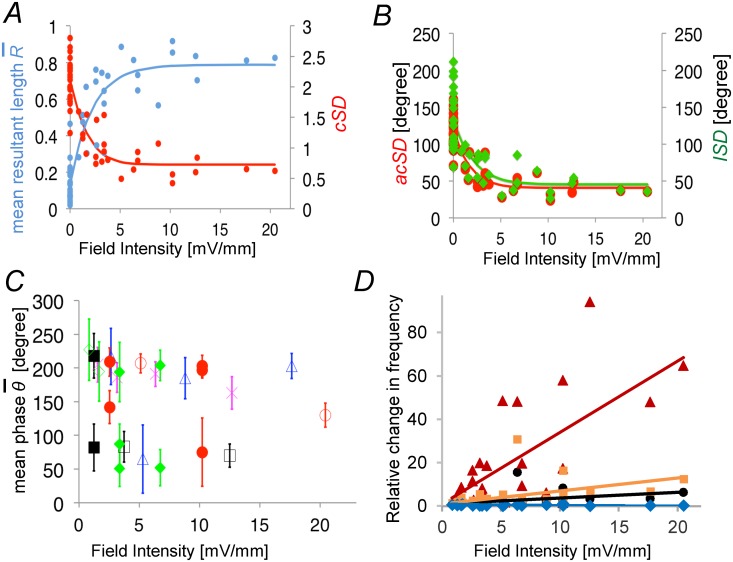
Dependence of the extent of entrainment and the relative change in frequency on the field strength. (*A*) Blue: The mean resultant length $\bar{R}$ plotted against field intensity (*FI*). The relation was fitted to an exponential function $\bar{R}$ = 0.13+0.65 (1-exp(-0.45*FI*)), (r = 0.92). Red: The circular standard deviation (*cSD* = sqrt(-2log($\bar{R}$)) plotted against field intensity. The relation was fitted to an exponential function *cSD* = 0.72 + 16.81×exp(-(*FI* +9.27)^2^/5.86^2^), (r = 0.89). 5 slices, 24 trials (4 Hz, 19 trials; 2 Hz, 4 trials; 1 Hz, 1 trial). (*B*) Red: The circular standard deviation in terms of angular degree (*acSD* = (180°/π)*cSD*) plotted against field intensity. The relation was fitted to an exponential function *acSD* = 40.76 + 1.13×10^5^×exp(-(*FI* +26.01)^2^/9.65^2^), (r = 0.89). Green: The linear standard deviation (*lSD*) plotted against field intensity. The relation was fitted to an exponential function *lSD* = 48.83 + 2.48×10^3^×exp(-(*FI* +15.43)^2^/-8.36^2^),(r = 0.74). Note that there was no noticeable change in the extent of entrainment within the intensity range of 5–22 mV/mm. (*C*) The mean phase *$\bar{\theta }$* plotted against field intensity. Marks of the same color represent data from the same slice preparation. Filled marks are used for the five cases in which electric field with the same intensity and frequency were applied. Note that the mean phase was different from one trial to the next. (*D*) The relative change in frequency of fast-Ca-transients during field stimulation plotted against field intensity. Orange: Relative change in frequency (RCF) during a preferred hemi-circle, that is a hemi-circle with 180° angular width with the center angle pointing toward the mean phase (*RCF* = 0.60*FI*+1, r^2^ = 0.46). Blue: Relative change in frequency during anti-preferred hemi-circle, the opposite hemi-circle of the field (*RCF* = exp(-0.13*FI*), r^2^ = -0.52). Red: The ratio of the relative change in frequency (*RRCF*) during preferred and anti-preferred hemi-circle (*RRCF* = 3.30*FI*+1, r^2^ = 0.46). Black: The mean relative change in frequency (*mRCF*) during full circle compared to the frequency in condition with no field stimulation (*mRCF* = 0.27*FI*+1, r^2^ = 0.13). 5 slices. 22 trials (4 Hz, 17 trials; 2 Hz, 4 trials; 1 Hz, 1 trial).

Fig. 8A shows $\bar{R}$ and the *cSD* calculated for transients during sinusoidal field stimulation plotted against field intensity. The value of $\bar{R}$ increased sharply from around 0.1 at zero field intensity to 0.8 at an intensity 5 mV/mm, then remained nearly constant above 5 mV/mm. For the data shown in Fig. 8A, the significance probability *P* for the Rayleigh test was smaller than 10^-6^ in all cases in which electrical field was applied. Even during the field with intensity of 0.84 mV/mm, the *P* value was smaller than 10^-6^.

Fig. 8B shows the *circular standard distribution in terms of angular degree* (*acSD*), calculated by multiplying the *cSD* by the factor 180°/π, plotted against field intensity. The *acSD* at a field intensity of 1.3 mV/mm was about 80°, implying that 68.3% of the fast-Ca-transients appeared within a range of 160° around the mean phase [37], while that at a field intensity of 20 mV/mm was about 45°, implying that 68.3% of the fast-Ca-transients appeared within a range of 90° around the mean phase.

In the example shown in Fig. 7, the mean phase at a field intensity of 12.5 mV/mm and 3.8 mV/mm was, respectively, 70° and 83°, whereas the mean phase at a field intensity of 1.3 mV/mm was 218°. There was a difference in mean frequency of about 140°. Fig. 8C shows the distribution of mean phase *$\bar{\theta }$* for fast-Ca-transients during field stimulations. In five cases in which electric field with the same intensity and frequency were applied, the mean phase was different from one trial to the next.

In previous studies [42, 43], we showed that rectangular-shaped extracellular electric fields with intensity greater than 40 mV/mm evoke spikes in pyramidal neurons. Although the field intensities used in the present study were lower than 40 mV/mm, it is possible that pyramidal neurons bathed in ACSF/4-AP containing 3.5–7.5 mM K^+^ ion generate spikes in response to much weaker electric field (< 40 mV/mm). If this was that case, then the entrainment was merely due to additional spikes triggered directly by the field stimulation not because the applied electric field affected the timing of spike generation. In order to test this possibility, we compared overall change in frequency of fast-Ca-transients during field stimulation with those during a preferred hemi-circle, that is a hemi-circle with 180° angular width with the center angle pointing toward the mean phase, and those occurred during anti-preferred hemi-circle, the hemi-circle opposite to the preferred hemi-circle. Fig. 8D shows that the mean frequency of fast-Ca-transients moderately increased during field stimulation as the intensity of the field become higher; below the field intensity of 5 mV/mm, the frequency during stimulation was 2.35 times as higher than that during no stimulation condition. If the field stimulation triggered additional transients only during the preferred hemi-circle, then the ratio of the frequency during preferred hemi-circle and anti-prepared hemi-circle should then be 3.7 (= 1+2(2.35–1)). However, the ratio of the frequency during preferred hemi-circle and anti-prepared hemi-circle increased steeply with the intensity of the field; 16.5 times at the field intensity of 5 mV/mm, much higher than expected from the above value indicating that the entrainment was not merely due to additional spikes triggered directly by the field stimulation but due to a shift of the timing of spike generation.

## Discussion

We have developed a method for monitoring local Ca-transients in the dendrites of a neuronal population in rat acute hippocampal slice preparations and examined the impact of sinusoidal electric fields. We found that the timing of spontaneously occurring fast-Ca-transients, probably associated with dendritic Na-spikes, in the distal apical dendrites of CA1 pyramidal neurons became mildly, but significantly, entrained to sub-threshold 1–4 Hz sinusoidal electric fields with an intensity as low as 0.84 mV/mm applied parallel to the soma-dendritic axis of the pyramidal neurons. The extent of entrainment increased with intensity within the intensity range below 5 mV/mm, but did not increase further over a wide range (5–20 mV/mm) of higher field intensities. These results suggest that population of pyramidal cells might be able to detect electric fields with biologically relevant intensity by modulating the timing of dendritic spikes.

### Experimental conditions for Ca measurements

To analyze the impact of an electric field on spontaneous activities of the dendrites, the slices were maintained in ACSF/4-AP solution. In normal ACSF, the soma and dendrites of the dye-stained neurons in hippocampal slice preparations rarely show Ca-transients likely be due to lower extracellular K^+^ concentration compared to in vivo situations and lack of neuromodulatory synaptic inputs. To mimic situations in vivo, we adjusted extracellular K^+^ concentration and added low concentration of 4-AP to the bathing solution. We used 4-AP because it has been reported that low concentration of 4-AP would stimulate release of neuromodulators from the presynaptic terminals [44, 45] probably by blocking Kv1 family voltage-gated K^+^ channels in the presynaptic terminals [46]. Low concentration of 4-AP has been used to induce synchronized epileptiform activities in rat hippocampal slices [47–51] and we tried to find a condition in which neurons show non-synchronous spontaneous activities by modulating the conditions. We found that spontaneously occurring fast-Ca-transients could be induced in the dendrites by adjusting the K^+^ concentration to 3.5–7.5 mM and raising concentrations of divalent cations than those used in previous studies. In our experimental condition, most of the fast-Ca-transients recorded from the dendritic region were characterized as the local type, and the global type of fast-Ca-transients detected only occasionally.

### Dendritic activities responsible for generating the fast-Ca-transients

Previous Ca imaging studies have provided several possible explanations for the generation of fast-Ca-transients, namely Na-dependent spikes [23, 52], spontaneous elementary-Ca-release-events [53, 54], Ca-waves [55], NMDA-spikes [56, 57], NMDA plateau potentials, and Ca-dependent spikes [58]. Sensitivity to TTX suggests that the fast-Ca-transients are due to generation of Na-dependent spikes in the dendrites.

The generation of fast spikes in the dendrites of hippocampal pyramidal neurons has been known for many years [19, 20, 23, 59, 60]. Early studies showed that Na-dependent spikes can be initiated synaptically at the cell body and propagate back into the apical dendrites [23]. Ca imaging studies [23, 52] showed that propagation is restricted to the shaft of the apical dendrites and fails to invade into finer dendrites. However, more recent studies showed that Na-dependent spikes can be initiated in the dendrites [58] not only in the shaft of the apical dendrites, but also in the tufts [61] and oblique dendrites [62]. The rise and decay time course of the Ca-transients associated with spikes in these studies are consistent with our conclusion that the generation of Na-dependent spikes gave rise to fast-Ca-transients.

Manita & Ross [53] reported that spontaneously occurring Ca-transients could be recorded from the dendrites of CA1 pyramidal cells in acute rat hippocampal slices using the same Ca indicator dye (OGB-1: 25–100 μM) used in our study. Based on the sensitivity to drugs that suppress Ca-release from intracellular Ca stores and insensitivity to TTX and ionotropic receptor antagonists, they concluded that the signal was caused by Ca-release from intracellular stores mainly by mobilization of IP_3_, and referred to these events as spontaneous elementary-Ca-release-events. Although the rise and decay times of the fast-Ca-transients in our study were similar to those of the elementary-Ca-release-events, the finding that they were blocked by application of TTX indicates that the signal was not due to elementary-Ca-release-events, but due to the generation of Na-dependent spikes. This, then, raises a question as to why elementary-Ca-release-events were not detected in our present study. Miyazaki et al. [63] have examined the developmental profile of elementary-Ca-release-events and reported that the relative amplitude of the elementary-Ca-release event compared to the Ca-transients due to back-propagating spikes is high in slices prepared from young animals (0.34 ± 0.02: P3–P15), but low in older animals (0.18 ± 0.02: P16–P40). It is likely that the signals due to elementary-Ca-release-events in our study were small compared to spike-related signals because we used old animals (P28–P56), and hence were not detected in our study.

### Intensity of the electric fields

We found that the entrainment of local-fast-Ca-transients became progressively evident as the intensity of the electric fields increased within a range lower than 5 mV/mm. The intensity of the electric fields during rhythmical EEG activity in behaving animals has been reported to be in this range, typically 2–4 mV/mm for theta oscillation with phase reversal from the str. pyramidale to the str. lacnosum-moleculare [1–5], 0.5 mV/mm for gamma oscillation with phase reversal at the border between the str. pyramidale and the str. radiatum [10], 1–2 mV/mm for ripples across the pyramidal cell layer [11], and 1.2 mV/mm for slow oscillations [3]. During a sharp wave, the field intensity across the pyramidal layer can be as large as 8–14 mV/mm [12]. Our findings therefore imply that naturally occurring electric fields during rhythmical EEG activities should be capable of modulating the collective activity of a neuronal population.

The threshold for the effects of electric fields on the activities of hippocampal neurons has been measured using electrophysiological techniques. Francis et al. [17] reported that a Gaussian-wave-form field pulse with an amplitude of 0.298 mV/mm (peak-to-peak) modulates ensemble firing in the CA1 area in rat hippocampal slices. Later, using intracellular recording technique for CA1 pyramidal neurons in rat hippocampal slices, Radman et al. [64] showed that a 30 Hz sinusoidal field with an intensity as low as 1 mV/mm gives rise to coherence of firing timing with the applied field. Deans et al. [18] showed that a sinusoidal field with an intensity of 0.5–1 mV/mm at 50 Hz has significant effects on the timing of CA3 pyramidal cell firing induced by application of kainic acid. Entrainment of the dendritic activities to the applied field may have been involved in the modulation of cell firing in these studies.

### Possible mechanisms for the entrainment

In our study, significant entrainment of fast-Ca-transient was induced by weak extracellular sinusoidal electric fields. In previous studies [42, 43], we reported that, in response to rectangular step field stimulation, the somatic region showed monophasic polarization, while the distal dendritic region showed biphasic polarization consisting of a fast polarization with a direction opposite to the somatic response, followed by a slow repolarization. The direction of the fast polarization was reversed at the middle of the str. radiatum about 160 μm from the str. pyramidale. In the present study, since we performed Ca imaging in the distal dendritic region more than 350 μm away from the str. pyramidale, the direction of the change in membrane potential of the dendrites should be depolarizing when the direction of the current for field stimulation was directed from the basal dendrites to the apical dendrites. Considering the time constant of responses for the rectangular step field at the dendrites (fast time constant 14.2 ± 1.2 msec and slow time constant 42.2 ± 2.8 msec in Akiyama et al., [43]), the peak of the depolarization in the apical dendrites should be delayed by less than 40 msec compared to the peak of the applied field. Then, if membrane depolarization alone determines the timing of spike generation, the timing of the fast transients would be delayed from the peak for the field by less than 40 msec. In case of 2 Hz sinusoidal field, and with the convention we use in spiral plot of our data, the mean phase $\bar{\theta }$during stimulation should be 90°-130°. Our result that the mean phase was rarely in this range and was different from trial to trial requires some other explanations for the entrainment. Kinetic states of ion channels distributed along the dendrites, and the propagation of spikes initiated at other parts of the cell are some of the factors that need to be considered.

### Functional significance of the entrainment

The extent of entrainment was mild ($\bar{R}$ around 0.8) over a wide range of physiologically-plausible field intensities with the width of the phase window within which the dendritic activities took place ranged 90°- 160° (*acSD* ranged 45°- 80°) over an intensity range of 5–20 mV/mm. It has been postulated that the phase of spike timing of hippocampal “place cells” with reference to the hippocampal theta wave may have functional significance in coding the position of an animal in the place field [65]. It may be reasonable to postulate that the output of pyramidal neurons during oscillating extracellular electric fields accompanying theta wave depends on the timing of synaptic inputs relative to the phase of the electric field. We expect that the interplay of a barrage of synaptic inputs on various parts of the dendrites and ongoing dendritic activities, the timing of which can be entrained to the extracellular oscillating electric field, determines the output of pyramidal neurons. The wide phase window of the entrained dendritic activity over a wide range of field intensities might provide a robust cellular basis for the phase-coding in neural systems [66].
